# *uORF-Tools*—Workflow for the determination of translation-regulatory upstream open reading frames

**DOI:** 10.1371/journal.pone.0222459

**Published:** 2019-09-12

**Authors:** Anica Scholz, Florian Eggenhofer, Rick Gelhausen, Björn Grüning, Kathi Zarnack, Bernhard Brüne, Rolf Backofen, Tobias Schmid

**Affiliations:** 1 Institute of Biochemistry I, Faculty of Medicine, Goethe-University Frankfurt, Frankfurt am Main, Germany; 2 Bioinformatics Group, Department of Computer Science, University of Freiburg, Freiburg, Germany; 3 Buchmann Institute for Molecular Life Sciences (BMLS), Goethe-University Frankfurt, Frankfurt am Main, Germany; 4 Centre for Biological Signalling Studies (BIOSS), University of Freiburg, Freiburg, Germany; University of British Columbia, CANADA

## Abstract

Ribosome profiling (ribo-seq) provides a means to analyze active translation by determining ribosome occupancy in a transcriptome-wide manner. The vast majority of ribosome protected fragments (RPFs) resides within the protein-coding sequence of mRNAs. However, commonly reads are also found within the transcript leader sequence (TLS) (aka 5’ untranslated region) preceding the main open reading frame (ORF), indicating the translation of regulatory upstream ORFs (uORFs). Here, we present a workflow for the identification of translation-regulatory uORFs. Specifically, *uORF-Tools* uses *Ribo-TISH* to identify uORFs within a given dataset and generates a uORF annotation file. In addition, a comprehensive human uORF annotation file, based on 35 ribo-seq files, is provided, which can serve as an alternative input file for the workflow. To assess the translation-regulatory activity of the uORFs, stimulus-induced changes in the ratio of the RPFs residing in the main ORFs relative to those found in the associated uORFs are determined. The resulting output file allows for the easy identification of candidate uORFs, which have translation-inhibitory effects on their associated main ORFs. *uORF-Tools* is available as a free and open *Snakemake* workflow at https://github.com/Biochemistry1-FFM/uORF-Tools. It is easily installed and all necessary tools are provided in a version-controlled manner, which also ensures lasting usability. *uORF-Tools* is designed for intuitive use and requires only limited computing times and resources.

## Introduction

Translation is a highly regulated cellular process, regulation occurring predominantly at the level of initiation [1]. Global translation is initiated in a cap-dependent manner, i.e. via binding of the cap-binding protein eukaryotic initiation factor 4E (eIF4E) to the 5’ 7-methyl-guanosine (m^7^G) cap present in all eukaryotic mRNAs and subsequent recruitment of the eIF4F initiation complex. As cap-dependent translation initiation depends on the availability of eIF4E, sequestration of the latter by eIF4E-binding proteins (4E-BPs), which are regulated by the central mTOR kinase, provides a means to efficiently control global translation [2]. In addition, there are numerous regulatory mechanisms that affect the translation of selected mRNAs only. Alternative modes of translational regulation commonly depend on *cis*-regulatory features within the 5’ untranslated region (UTR) of the respective mRNAs, e.g. specific sequences or secondary structures [3]. Alternative modes of translational regulation, such as internal ribosome entry site (IRES)- and upstream open reading frame (uORF)-dependent initiation, are of major importance under stress conditions, when global translation is inhibited, yet the synthesis of certain proteins needs to be sustained [4–5].

The analysis of translational changes was revolutionized by the development of the ribosome profiling (ribo-seq) technology, where actively translated regions are determined across the entire transcriptome by selective sequencing of ribosome protected footprints (RPFs) [6]. Sequencing reads in ribo-seq analyses are predominantly mapped to the protein-coding regions. Yet, while the 3’UTRs of transcripts usually lack RPFs, they are commonly observed in the 5’UTRs. Such actively translated regions are indicators for the presence of upstream open reading frames (uORFs), which represent short, peptide-coding sequences characterized by a start codon with an in-frame stop codon. Consequently, 5’UTRs are also referred to as transcript leader sequences (TLS) [7]. With respect to their function, uORFs have been shown to affect the translation of associated main ORFs. While there are cases in which translation of a uORF positively affects the translation of the main ORF, for the most part efficient translation of a uORF is considered to restrict the translation of the respective main ORF [8]. Of note, uORFs have been shown to play a prominent role during the integrated stress response (ISR), an adaptive response to various stress conditions aiming at restoring cellular homeostasis. During the ISR the translation initiation factor eIF2α is phosphorylated by protein kinase R (PKR), PKR-like endoplasmic reticulum stress (PERK), heme-regulated inhibitor (HRI), or general control non-repressible 2 (GCN2) kinases in response to stress conditions such as amino acid deprivation, viral infection, heme deprivation, and endoplasmic reticulum stress [9]. Phosphorylation of eIF2α reduces global translation and at the same time enhances translation of selected, uORF-bearing mRNAs to allow for adaptation [10]. Such stress adaptive mechanisms are of major importance in a number of disease states including cancer and inflammation [11].

There are various strategies to determine the presence of uORFs either based on sequence features within the TLS [12], or using experimental ribo-seq data to identify actively translated ORFs including uORFs (*ORFscore* [13]; *RiboTaper* [14]; *RiboLace* [15]; *PRICE* [16]; *RibORF* [17]; *sORF*.*org* [18]; *RiboCode* [19]; *RiboWave* [20]). With the present workflow, we aim to provide a pipeline that allows for the identification of differentially translated uORFs, which may regulate the translation of the associated main ORFs. Using ribosome profiling data, *uORF-Tools* determines the experiment-specific, differentially translated uORFs and compares their translation with the translation of the respective main ORFs. While a uORF annotation file is generated for each individual experiment using *Ribo-TISH* [21], a comprehensive human uORF annotation file, based on 35 data sets from nine human ribosome profiling data series, is also provided to allow for a comprehensive assessment of the translation regulatory impact of uORFs.

## Implementation and workflow

### Implementation

*uORF-Tools* is provided as a free and open workflow and can be downloaded from https://github.com/Biochemistry1-FFM/uORF-Tools. It is based on *Snakemake* [22] and automatically installs all tool dependencies in a version-controlled manner via *bioconda* [23]. The workflow can be run locally or in a cluster environment.

### Workflow

**Fig 1:**
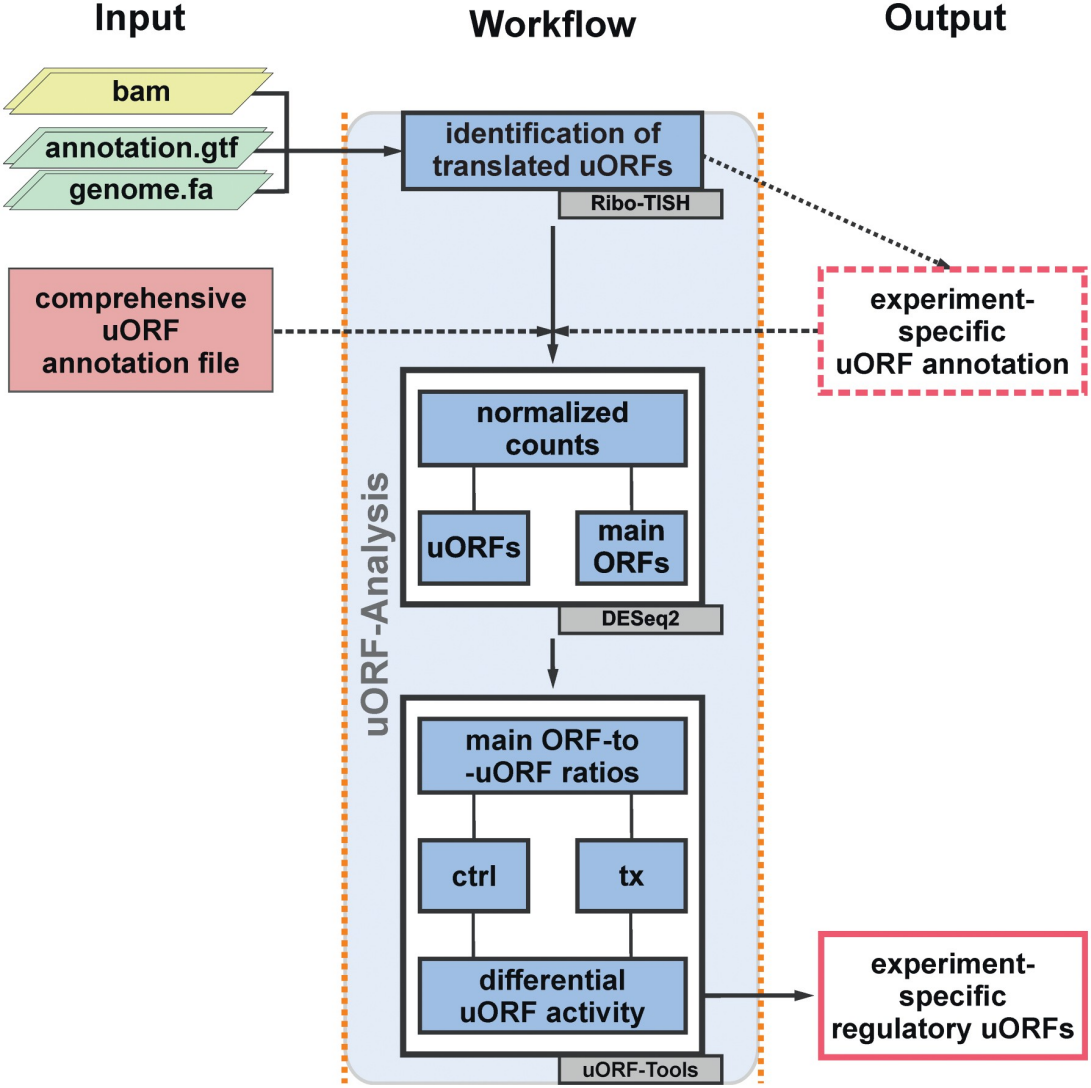
*uORF-Tools*—Workflow for the determination of translation-regulatory uORFs. Required input is shown on the left, a simplified depiction of processing in the center, and results on the right. (ctrl: control; tx: treatment).

*uORF-Tools* is designed to receive bam files of ribosome profiling data sets as input (Fig 1). In addition, the workflow requires a genome fasta file and an annotation gtf file.

Initially, *uORF-Tools* generates a new genome annotation file, which is used in the subsequent steps of the workflow. For practical reasons, this annotation file contains only the validated or manually annotated (confidence levels 1 and 2 in Gencode) (www.gencodegenes.org) longest protein coding transcript variants. Based on the provided input bam files and the generated genome annotation file, an experiment-specific uORF annotation file is then generated using *Ribo-TISH* [21]. Specifically, *Ribo-TISH* identifies translation initiation sites within ribo-seq data and uses this information to determine ORFs, i.e. regular ORFs as well as uORFs. Default settings in uORF-Tools use the canonical start codon ATG only, yet users can allow for the use of alternative start codons as well. Furthermore, as uORFs are generally considered to be short, peptide-coding ORFs, a maximal length of 400 nt was set as upper size limit within the uORF-Tools pipeline for the identification of uORFs. The minimal size limit was set to 9 nt to ensure that the potential uORFs contain at least one codon on top of the required start and stop codons [24]. To allow for an even broader characterization of potentially active uORFs, a comprehensive human uORF annotation file (based on hg38), based on 35 ribo-seq data sets, is provided with the package (for details see S1 Table). Among other information, this file contains the exact coordinates of all uORFs (designated as ORFs in the annotation file), as well as their lengths. To use this comprehensive instead of the experiment-specific annotation file, the former needs to be selected by including its file path (uORF-Tools/comprehensive_annotation/uORF_annotation_hg38.csv) in the config.yaml file before starting the *uORF-Tools* workflow. Using uORF and genome annotation files, *uORF-Tools* creates one count file containing all reads that correspond to coding sequences (CDS) of the longest protein coding transcripts, i.e. main ORFs, and another count file which contains only reads that correspond to uORFs. To control for differences in library sizes, the count data are subsequently normalized using size factors calculated for all input libraries with *DESeq2* [25]. To determine the relative translation of a main ORF, counts of the main ORF are normalized to the corresponding uORF counts. In order to assess if the main ORF-to-uORF ratios are altered in response to a stimulus, the impact of uORFs on downstream translation is determined by comparing the main ORF-to-uORF ratios between different conditions. A stimulus-dependent increase in the ratios indicates enhanced translation of the main ORF, i.e. reduced repression by the respective uORF, conversely a decrease in the ratios indicates that an inhibitory uORF becomes more active. Of note, no translational efficiencies are determined and needed in the uORF-Tools pipeline, since both main ORF and uORF ribo-seq reads would be normalized to the same transcript abundance, which would be eliminated during the calculation of the main ORF-to-uORF ratios. We therefore decided to compare ribosome profiling reads only to minimize computing requirements. Along the same lines, *uORF-Tools* is designed to take bam files, i.e. processed ribosome profiling data. Nevertheless, we also provide a pre-processing pipeline (S1 File) to allow for the use of yet unprocessed fastq files.

## Results and discussion

*uORF-Tools* is provided as a readily deployable *Snakemake* workflow, which comes with extensive documentation (S1 File). Running *uORF-Tools* on 8 test data sets, i.e. 4 replicates of control and thapsigargin-treated HEK293 cells, with about 0.36 to 4.7 million reads per file on a consumer grade laptop (Intel^®^ Core^™^ i5-8265U, 256 GB NVMe-SSD, 16 GB RAM) running Ubuntu 18.04.2 LTS required as little as 1.5 hours for a complete analysis. The input data and the utilized tools are clearly defined and enable reproducible analyses (Fig 1; S1 File). Using an annotation.gtf and a genome.fa file obtained from Gencode (gencode.v28.annotation.gtf and GRCh38.p12.genome.fa), the analysis of the provided 8 test data sets (available at: ftp://biftp.informatik.uni-freiburg.de/pub/uORF-Tools/bam.tar.gz) identified 939 uORFs. In contrast, the provided, comprehensive uORF annotation file contains 1933 uORFs (Table 1, S2 Table). Interestingly, when the comprehensive annotation file was used in the analysis of the test data set, only 55 of the additional uORFs did not contain any RPF counts (S3 Table). This is likely due to the fact that the *Ribo-TISH* criteria for the identification of a uORF might prevent the comprehensive annotation of all translated uORFs given datasets with lower quality are analyzed. In fact, some of the uORFs showing the strongest impact on the translation of the downstream main ORF were not identified in the experiment-specific uORF annotation.

**Table 1:** Comparison of the performance of *uORF-Tools* for the 8 test data sets (GSE103719) using either the experiment-specific or the comprehensive annotation files.

	experiment-specific annotation file ^a^	comprehensive annotation file
identified uORFs	939	1933
mean length—main ORFs	1509	1575
mean length—uORFs	38	40
translation-inhibitory uORFs in test data set ^b^	47	94

(^a^ using 8 test data sets (GSE103719);

^b^ 5% quantile of strongest changes in main ORF-to-uORF ratios)

**Fig 2:**
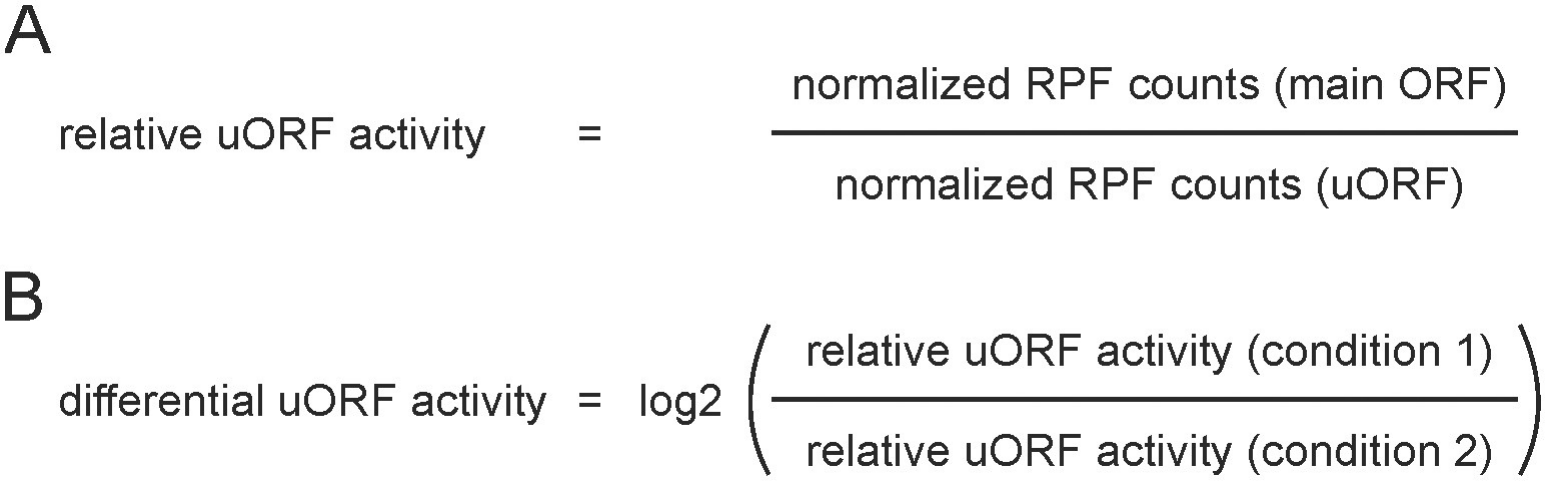
Calculation of relative uORF translation. (A) Relative uORF translation is determined for each experimental condition as ratio of the normalized ribosome protected fragment (RPF) counts of a specific main ORF relative the normalized RPF counts of the respective uORF. (B) Stimulus-dependent, differential uORF translation is then calculated as the log2 fold change of the ratio of the relative uORF translation of treatment (condition 1) vs. control (condition 2).

To assess how the translation of the main ORFs might be affected by the uORFs, main ORF read counts were initially normalized to those of the associated uORFs (Fig 2A). This yielded mean ratios of 23.42 ± 3.74 or 26.48 ± 3.85 for the control and 27.62 ± 8.86 or 29.85 ± 8.27 for the thapsigargin-treated samples, based on the experiment-specific or the comprehensive annotations files, respectively.

Furthermore, the main ORFs of the uORF-bearing transcripts were generally much longer (mean lengths 1509 and 1575 nt, based on experiment-specific and comprehensive annotation files, respectively) than the uORFs (mean length 38 and 40 nt, based on experiment-specific and comprehensive annotation files, respectively) (Table 1). Of note, the mean uORF lengths in either uORF annotation file were similar to the previously proposed median length of 48 nt across 11,649 predicted human uORFs [24].

**Fig 3:**
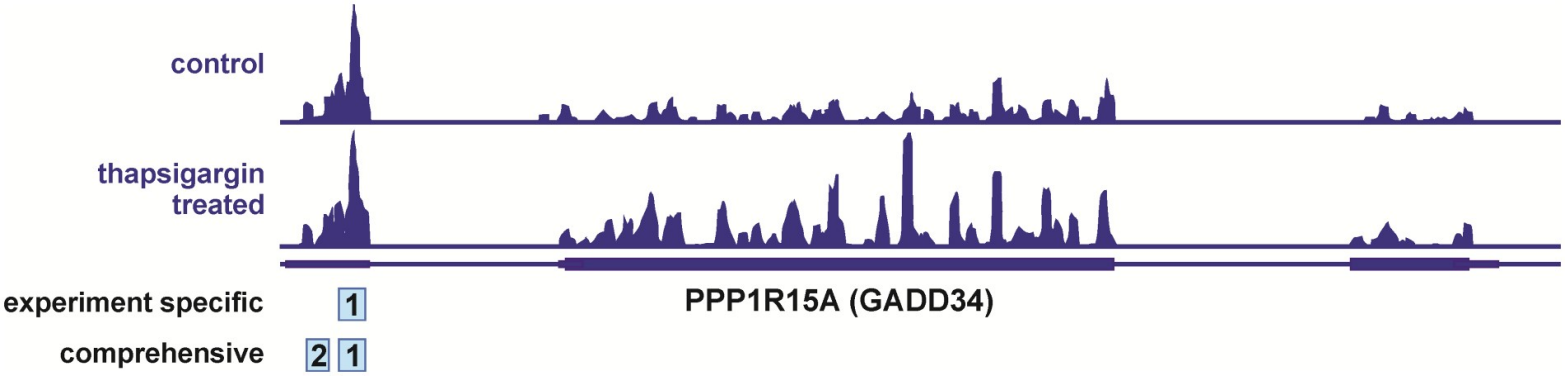
Distribution of RPF reads on the PPP1R15A (GADD34) transcript. Reads of control (*upper panel*) or thapsigargin-treated (*lower panel*) HEK293 from data set GSE103719 are shown. uORFs annotated either in the experiment-specific (1) or the comprehensive annotation file (1 and 2) within *uORF-Tools* are marked.

Subsequent calculation of the stimulus-dependent changes in main ORF-to-uORF ratios (Fig 2B), provides a means to easily identify uORFs inversely correlating with their associated main ORFs with respect to the transcript-specific ribosome occupancy. Owing to the major length differences between uORFs and their associated main ORFs, the dynamic range for changes in ribo-seq reads is higher on the side of the main ORFs. Consequently, changes in the ratios can be expected to be strongly influenced by changes in main ORF translation (compare Fig 3).

In the case of the analyzed test data sets, the 5% quantile of the strongest changes in differential uORF translation was comprised of 47 transcripts based on the experiment-specific uORF annotation file, as compared to 94 transcripts in the case of the comprehensive uORF annotation file (Table 1, S3 Table). These differences underscore that it is advantageous to use comprehensive uORF annotations rather than experiment-specific ones only, as this might e.g. overcome low numbers of annotated uORFs due to ribo-seq analyses of either poor quality or containing low read numbers.

Along these lines, the experiment-specific annotation file identified only one uORF (uORF 1: 163–243 nt, 26 amino acids) within the TLS of the classical ISR target protein phosphatase 1 regulatory subunit 15A (PPP1R15A, aka growth arrest and DNA-damage-inducible 34 (GADD34)), whereas both published uORFs (uORF 2: 64–132 nt, 22 amino acids and uORF 1: 163–243 nt, 26 amino acids) [26] were found using the comprehensive uORF annotation file. The uORFs within the TLS of PPP1R15A were exactly annotated as previously published. They were further found to be highly translated, as becomes already apparent when looking at the relative RPF peak heights of the uORFs relative to the main ORF, wherein a shift from a largely uORF-biased distribution under control conditions to more RPF reads in the thapsigargin group can be seen (Fig 3).

Quantitative analyses of the main ORF-to-uORF ratios revealed that the 5% quantile of strongest changes showed differential uORF translations of log2FC > |1.99| / |1.88| (based on the experiment-specific and the comprehensive annotation file, respectively) (S3 Table).

In the case of PPP1R15A, translation under control conditions (main ORF-to-uORF ratios: uORF 1 = 5.51; uORF 2 = 9.40), shifted towards the main ORF under thapsigargin treatment (main ORF-to-uORF ratios: uORF 1 = 21.61; uORF 2 = 80.10). Specifically, the main ORF-to-uORF ratio of PPP1R15A displayed a log2FC increase of 1.87 and 4.26 for uORF 1 and uORF 2, respectively. This indicates that the translational repression under control conditions is relieved during the integrated stress response (ISR) and consequently the translation of the PPP1R15A main ORF increases, as previously reported. In line with previous reports, our data further suggest that uORF 2 translation is more important for the regulation of the translation of the downstream main ORF of PPP1R15A [8, 26]. As a side note, *uORF-Tools* determines the impact of each uORF on the translation of the associated main ORF independently. Yet, if multiple uORFs exist within the same transcript, the impact of different uORFs on the regulation of the same main ORF can be easily compared in the output as all uORF IDs contain unique specifier appended to the transcript ID (S2 and S3 Tables).

Corroborating the concept that the translation of otherwise uORF-repressed main ORFs is elevated during the ISR [27], only 6 of the 94 candidates within the 5% quantile of strongest changes, as identified using the comprehensive annotation file, showed reduced main ORF-to-uORF ratios, i.e. enhanced translational repression by the uORF. Furthermore, 675 candidates had main ORF-to-uORF ratios log2FC < 0 (73 candidates log2FC < -1), while 1245 had log2FC > 0 (411 candidates log2FC > 1). Along the same lines, the mean of all reduced main ORF-to-uORF ratios was log2FC = -0.50 and the mean for all elevated ones log2FC = 0.83 (S3 Table). All of these findings support the notion that thapsigargin relieves uORF-mediated translational repression of specific targets.

In addition to the identification of translation-inhibitory uORFs, the output file also contains uORFs that are regulated in the same direction as their associated main ORFs, which may indicate a translation-supportive function of the respective uORFs. The candidates within the 5% quantile of least changes display main ORF-to-uORF ratios log2FC < |0.05|. It should be noted that, while the translation-inhibitory uORFs are easily identified with *uORF-Tools*, the unambiguous identification of translation-supporting uORFs would require additional information. For example, translation efficiencies could be used to analyze whether main ORF-uORF pairs with unaltered ratios in fact exhibit homo-directional changes or whether these pairs are not regulated at all.

In addition to the stimulus-dependent changes in main ORF-to-uORF ratios, as indicators for the impact of the uORFs on downstream translation, the output folder contains the files ribo_norm_CDS_reads.csv and ribo_norm_uORFs_reads.csv with read counts for uORFs and main ORFs under all conditions tested. This will be informative for the assessment of the translational status of the individual transcripts and, thus, the potential relevance of the determined changes in a given data set.

## Availability and future directions

The *uORF-Tools* workflow is provided as free and open software (https://github.com/Biochemistry1-FFM/uORF-Tools), which can be easily deployed with all version-controlled dependencies. Bam files of the used 8 test data sets (GSE103719 [28]) are available at ftp://biftp.informatik.uni-freiburg.de/pub/uORF-Tools/bam.tar.gz. Extensive documentation of the workflow is provided with the software and supplied in the supporting information (S1 File).

*uORF-Tools* generates an intuitive, easy to interpret output file, containing the stimulus-dependent changes in main ORF-to-uORF ratios as an indicator for uORFs that negatively regulate the translation of their associated main ORFs. In addition, *uORF-Tools* provides a comprehensive human uORF annotation file based on 35 ribosome profiling data sets (S1 Table), which appeared superior to experiment-specific uORF annotation files, with respect to the identification of translation-regulatory uORFs. Future updates will incorporate comprehensive uORF annotations files for additional species.

Even with limited computing resources, *uORF-Tools* is a fast software solution and a valuable addition to the portfolio of methods for researchers interested in the function of uORFs.

## Supporting information

S1 File A more detailed description of the *uORF-Tools* workflow and its implementation. (PDF)

S1 Table Ribo-seq data series used for the generation of the comprehensive human uORF annotation file. 35 data sets from nine different data series each included ribo-seq and associated RNA-seq data.
(PDF)

S2 Table Lists of uORFs in the comprehensive and in the experiment-specific annotation files. (XLSX)

S3 Table Output files generated using the comprehensive or the experiment-specific annotation files. (XLSX)
