# Supplementary material for: uORF-Tools—Workflow for the determination of translation-regulatory upstream open reading frames
Source: PLoS One. 2019 Sep 12;14(9):e0222459. doi: 10.1371/journal.pone.0222459 (PMC6742470; doi:10.1371/journal.pone.0222459)
Supplement: S1 Table — 35 data sets from nine different data series each included ribo-seq and associated RNA-seq data. (PDF) [file pone.0222459.s002.pdf]

S1 Table. Ribo-seq data series used for the generation of the comprehensive human uORF annotation file. Data sets came from nine different data series and consisted of variable numbers of treatments and replicates. 35 data sets of individual samples each included ribo-seq and associated RNA-seq data.

Data series #	Date series ID	Data set (ribo-seq IDs)
1	GSE103719	GSM2779669 / GSM2779661<br>GSM2779670 / GSM2779662<br>GSM2779671 / GSM2779663<br>GSM2779672 / GSM2779664<br>GSM2779673 / GSM2779665<br>GSM2779674 / GSM2779666<br>GSM2779675 / GSM2779667<br>GSM2779676 / GSM2779668
2	GSE66929	GSM1634443 / GSM1632189<br>GSM1634445 / GSM1632191<br>GSM1634449 / GSM1632193
	GSE96716	GSM2538903 / GSM2538901<br>GSM2538904 / GSM2538902
4	GSE42509	GSM1047584 / GSM1041191<br>GSM1047585 / GSM1041192<br>GSM1047586 / GSM1041193<br>GSM1047587 / GSM1041194<br>GSM1047591 / GSM1041199
5	GSE69602	GSM1704511 / GSM1704559<br>GSM1704513 / GSM1704561<br>GSM1704523 / GSM1704571<br>GSM1704525 / GSM1704573
6	GSE56924	GSM1371443 / GSM1371395<br>GSM1371449 / GSM1371401<br>GSM1371455 / GSM1371407<br>GSM1371461 / GSM1371413
7	GSE96714	GSM2538884 / GSM2538879
8	GSE114636	GSM3146275 / GSM3146283<br>GSM3146276 / GSM3146284
9	GSE98623	GSM2602082 / GSM2602073<br>GSM2602083 / GSM2602074<br>GSM2602084 / GSM2602075<br>GSM2602085 /GSM2602076<br>GSM2602086 / GSM2602077<br>GSM2602087 / GSM2602078
